# Neuroprotective Effects of Polysaccharides and Gallic Acid from *Amauroderma rugosum* against 6-OHDA-Induced Toxicity in SH-SY5Y Cells

**DOI:** 10.3390/molecules29050953

**Published:** 2024-02-22

**Authors:** Panthakarn Rangsinth, Nattaporn Pattarachotanant, Wen Wang, Polly Ho-Ting Shiu, Chengwen Zheng, Renkai Li, Tewin Tencomnao, Siriporn Chuchawankul, Anchalee Prasansuklab, Timothy Man-Yau Cheung, Jingjing Li, George Pak-Heng Leung

**Affiliations:** 1 Department of Pharmacology and Pharmacy, The University of Hong Kong, Hong Kong SAR, China; ptkrs@hku.hk (P.R.); skye99@hku.hk (W.W.); u3009114@connect.hku.hk (P.H.-T.S.); u3006786@connect.hku.hk (C.Z.); rkli@hku.hk (R.L.); 2 Department of Clinical Chemistry, Faculty of Allied Health Sciences, Chulalongkorn University, Bangkok 10330, Thailand; nat.ahs11@gmail.com (N.P.); tewin.t@chula.ac.th (T.T.); 3 Department of Transfusion Medicine and Clinical Microbiology, Faculty of Allied Health Sciences, Chulalongkorn University, Bangkok 10330, Thailand; siriporn.ch@chula.ac.th; 4 College of Public Health Sciences, Chulalongkorn University, Bangkok 10330, Thailand; anchalee.pr@chula.ac.th; 5 Tian Ran Healthcare Limited, Hong Kong SAR, China; timothyc@mytianran.com; 6 Department of Rehabilitation Sciences, Faculty of Health and Social Sciences, Hong Kong Polytechnic University, Hong Kong SAR, China

**Keywords:** *Amauroderma rugosum*, antioxidant, neuroprotective, gallic acid, polysaccharides

## Abstract

The pharmacological activity and medicinal significance of *Amauroderma rugosum* (AR) have rarely been documented. We examined the antioxidant and neuroprotective effects of AR on 6-hydroxydopamine (6-OHDA)-induced neurotoxicity in an SH-SY5Y human neuroblastoma cell model of Parkinson’s disease (PD) and explored the active ingredients responsible for these effects. The results showed that the AR aqueous extract could scavenge reactive oxygen species and reduce SH-SY5Y cell death induced by 6-OHDA. In addition, the AR aqueous extract increased the survival of *Caenorhabditis elegans* upon juglone-induced toxicity. Among the constituents of AR, only polysaccharides and gallic acid exhibited antioxidant and neuroprotective effects. The AR aqueous extract reduced apoptosis and increased the expression of phospho-Akt, phospho-mTOR, phospho-MEK, phospho-ERK, and superoxide dismutase-1 in 6-OHDA-treated SH-SY5Y cells. The polysaccharide-rich AR extract was slightly more potent than the aqueous AR extract; however, it did not affect the expression of phospho-Akt or phospho-mTOR. In conclusion, the AR aqueous extract possessed antioxidant and neuroprotective properties against 6-OHDA-induced toxicity in SH-SY5Y cells. The mechanism of action involves the upregulation of the Akt/mTOR and MEK/ERK-dependent pathways. These findings indicate the potential utility of AR and its active ingredients in preventing or treating neurodegenerative disorders associated with oxidative stress such as PD.

## 1. Introduction

Parkinson’s disease (PD) is the second most prevalent neurodegenerative disorder globally, with an increasing incidence among individuals aged over 60 years [1]. PD is characterized by the gradual and specific degeneration of dopaminergic neurons located in the substantia nigra pars compacta. This degeneration results in significant impairments in movement such as postural instability, involuntary tremors, muscle stiffness, and bradykinesia. The existing interventions for PD include dopaminergic replacement therapy and deep-brain stimulation therapy [2]. However, they do not impede or decelerate PD advancement. Therefore, developing innovative pharmaceutical products to reverse the progression of PD is of paramount importance.

Despite the ongoing uncertainty surrounding the pathogenic mechanisms underlying PD, a growing body of scientific evidence supports the notion that the degeneration of dopaminergic neurons is closely linked to cell injury generated by oxidative stress [3]. The excessive build-up of reactive oxygen species (ROS) within dopaminergic neurons can potentially harm many biological components such as lipids, proteins, and nucleic acids. This, in turn, triggers an intracellular inflammatory response, leading to mitochondrial dysfunction, cellular damage, and, ultimately, apoptosis. Hence, the mitigation of oxidative damage in dopaminergic neurons as a potential strategy for treating PD has garnered attention [4].

*Amauroderma rugosum* (AR) is a basidiomycete belonging to the Ganodermataceae family. Although people in China and South Asia frequently consume AR, studies investigating its potential health benefits and medicinal properties are scarce. Our previous investigation provided evidence that the AR extract protects PC12 cells from oxidative stress, mitochondrial malfunction, and apoptosis induced by 6-hydroxydopamine (6-OHDA) [5]. Furthermore, the AR extract directly scavenges ROS, downregulates pro-apoptotic proteins, and upregulates signaling pathways dependent on Akt/mTOR and MEK/ERK. Our study was the first to document the plausible neuroprotective effects of AR.

PC12 cells undergo neuronal differentiation upon exposure to nerve growth factors. Consequently, they produce neurotransmitters such as dopamine, noradrenaline, and acetylcholine [6]. It is noteworthy that PC12 cells are derived from rat adrenal pheochromocytomas. To overcome this limitation, SH-SY5Y cells are frequently used as in vitro models of neurodegenerative disorders because of their human neuroblastoma origin. Upon exposure to retinoic acid, SH-SY5Y cells undergo differentiation and acquire characteristics resembling dopaminergic neurons [7]. To enhance the validity of our prior research outcomes involving PC12 cells, we investigated the neuroprotective effect of AR on the toxicity induced by 6-OHDA in SH-SY5Y cells. More importantly, we aimed to investigate the active components responsible for the neuroprotective effects of AR.

## 2. Results

### 2.1. Chemical Compositions of AR Water Extract (ARW) and Polysaccharide-Rich AR Extract (ARP)

The chemical assay results indicated that the total polysaccharide contents in the ARW and ARP samples were 118.2 ± 12.44 and 238.52 ± 23.08 mg GE/g, respectively. The total triterpene concentration in the ARW and ARP samples was 15.15 ± 3.15 mg OA/g and 8.88 ± 0.76 mg OA/g, respectively. The total phenolic compound concentration in the ARW and ARP samples was 30.39 ± 6.66 mg GAE/g and 17.47 ± 4.05 mg GAE/g, respectively. The flavonoid content in the ARW and ARP samples was 12.61 ± 2.78 mg QE/g and 6.06 ± 0.43 mg QE/g, respectively. The total protein concentrations in the ARW and ARP samples were 98.43 ± 2.47 mg BSA/g and 51.31 ± 2.18 mg BSA/g, respectively.

### 2.2. Antioxidant Capacities of ARW, ARP, and the Different Components in the ARW

The free radical-scavenging capacity (SC) of ARW toward the DPPH• free radicals was positively correlated with its concentration. The SC_50_ value of ARW was 41.19 µg/mL.

To determine the active ingredient responsible for the antioxidant effects of ARW, DPPH assays were performed on the chemical components of ARW, as described in a previous study [8]. Of the components examined, only gallic acid demonstrated a scavenging effect on the DPPH• free radicals, with an SC_50_ value of 7.429 µM.). The DPPH assay also showed that ARP could scavenge DPPH• free radicals in a concentration-dependent manner, with an SC_50_ value of 12.88 µg/mL. Vitamin C, which served as the positive control, scavenged DPPH free radicals with an SC_50_ of 19.32 ± 0.47 µM.

### 2.3. Protective Effects on ARW, ARP, and Gallic Acid on 6-OHDA-Induced SH-SY5Y Cell Death

**Figure 1:**
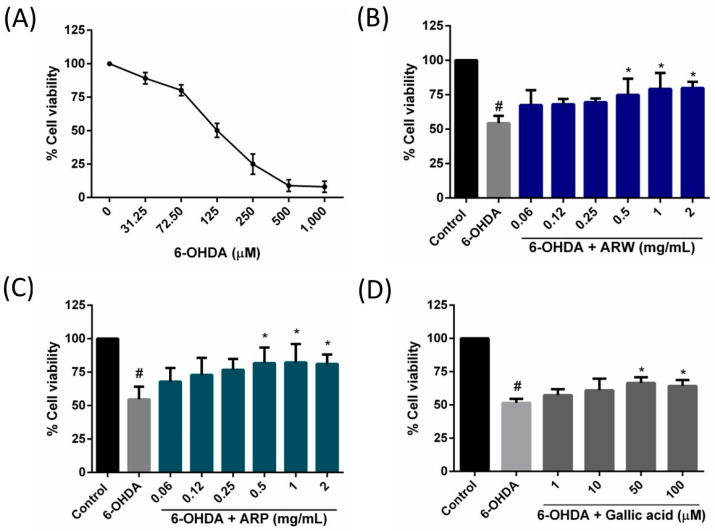
Effects of ARW, ARP, and gallic acid on protecting SH-SY5Y cells against 6-OHDA-induced cytotoxicity. (**A**) SH-SY5Y cells were treated with various concentrations of 6-OHDA for 24 h to evaluate their cytotoxicity. SH-SY5Y cell viability was then measured using the MTT assay. After obtaining the concentration-dependent response curve of 6-OHDA, the cells were pre-incubated with different concentrations of (**B**) ARW, (**C**) ARP, and (**D**) gallic acid for 2 h, followed by treatment with 125 µM 6-OHDA for 24 h. Untreated cells served as controls. Data are presented as the percentage relative to the control group values (mean ± SD of three independent experiments). # *p* < 0.05 indicates a statistically significant difference compared to the control group. * *p* < 0.05 indicates a statistically significant difference compared to the 6-OHDA-treated group.

In this study, SH-SY5Y cells were employed as a cellular model to investigate the neuroprotective properties of ARW, ARP, and gallic acid. First, the cytotoxic properties of ARW, ARP, and gallic acid were assessed by subjecting SH-SY5Y cells to different concentrations of these substances (0.06–2 mg/mL for ARW and ARP; 1–100 µM for gallic acid) for 24 h. After treatment, the results of the MTT assay showed that ARW, ARP, and gallic acid were not toxic. The cell viability was 102.5 ± 5.6%, 104.2 ± 4.7%, and 100.5 ± 2.3% when the SH-SY5Y cells were incubated with 2 mg/mL of ARW, 2 mg/mL of ARP, and 100 µM of gallic acid, respectively. Therefore, these concentration ranges were used in subsequent experiments. The concentration-dependent toxicity of 6-OHDA was observed in the SH-SY5Y cells (Figure 1A). Upon exposure to 125 μM of 6-OHDA, the viability of the SH-SY5Y cells decreased to 50.17 ± 5.19%. ARW, ARP, and gallic acid reduced 6-OHDA-induced cell death in a concentration-dependent manner (Figure 1B–D). When administered at 0.5 mg/mL, ARW and ARP increased the proportion of viable 6-OHDA-treated cells from 54.35 ± 5.26% to 74.81 ± 6.84% and from 54.67 ± 9.45% to 81.72 ± 5.85%, respectively. The application of gallic acid at concentrations below 10 µM had no effect, but concentrations exceeding 50 µM had a protective effect on 6-OHDA-treated SH-SY5Y cells, resulting in a notable increase in cell viability from 51.55 ± 2.96% to 66.47 ± 4.30% at 50 µM.

### 2.4. In Vitro and In Vivo Antioxidant Activities of ARW and ARP

**Figure 2:**
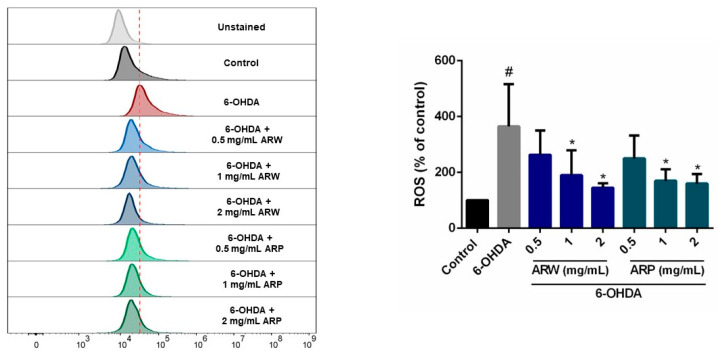
Effects of ARW and ARP on intracellular reactive oxygen species levels in SH-SY5Y cells. SH-SY5Y cells were pre-treated with different concentrations of ARW, ARP, and gallic acid for 2 h and then treated with or without 125 μM of 6-OHDA for 24 h. Untreated cells served as the control. SH-SY5Y cells were subjected to CM-H_2_DCFDA staining and the fluorescence signals were quantified using flow cytometry analysis. Data are presented as a percentage of the control group values (mean ± SD of three independent experiments). # *p* < 0.05 indicates a statistically significant difference compared to the control group. * *p* < 0.05 indicates a statistically significant difference compared to the 6-OHDA-treated group.

The in vitro antioxidant properties of ARW and ARP were investigated in SH-SY5Y cells treated with 6-OHDA. The intracellular production of ROS was indicated by the green fluorescent signal emitted by the CM-H_2_DCFDA probe. Flow cytometry revealed a 265% increase in ROS levels in SH-SY5Y cells induced by 6-OHDA. However, the application of 6-OHDA only resulted in a modest elevation in ROS levels, with an increase of 45% and 60% when the cells were pre-treated with 2 mg/mL of ARW and ARP, respectively (Figure 2).

**Figure 3:**
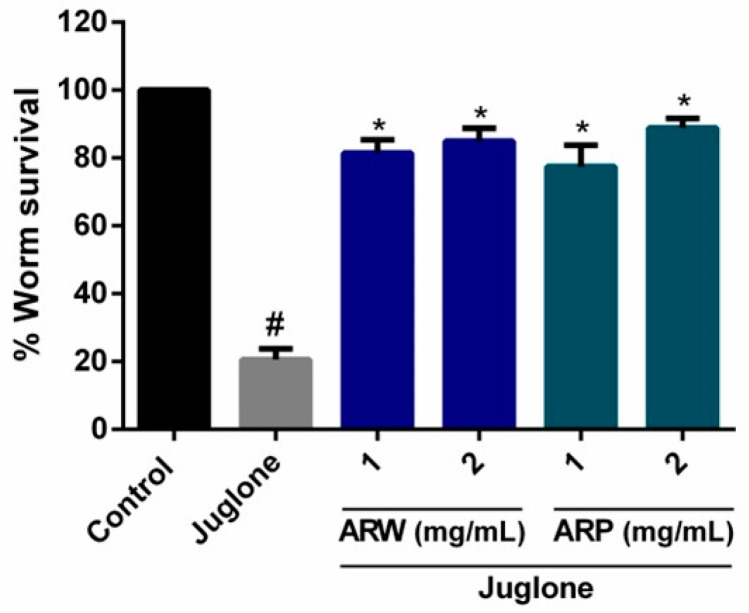
Effects of ARW and ARP on *Caenorhabditis elegans* survival. Oxidative stress in *C. elegans* was induced by treatment with 80 µM juglone for 24 h. The survival rate of the worms was calculated. The experiment was conducted using a minimum of 100 worms per group. All values are reported as the mean ± SEM. # *p* < 0.05 indicates a statistically significant difference compared to the control group. * *p* < 0.05 indicates a statistically significant difference compared to the 6-OHDA-treated group.

The protective effects of ARW and ARP were also assessed in *Caenorhabditis elegans*. Administration of ARW and ARP at 2 mg/mL did not significantly affect the longevity of *C. elegans* (Table 1), indicating that ARW and ARP were non-toxic. Juglone, a yellow-pigmented pro-oxidant derived from the plant species *Juglans regia*, is frequently used to induce mortality in *C. elegans*. The mechanism of action of juglone involves ROS generation. Only 20.52 ± 3.26% of the worms survived after exposure to 80 μM of juglone. However, the survival rates of the worms dramatically increased to 84.91 ± 3.83% and 88.90 ± 2.73% when they were pre-treated with 2 mg/mL of ARW and ARP, respectively (Figure 3).

### 2.5. Anti-Apoptotic Effects of ARW and ARP in SH-SY5Y Cells

**Figure 4:**
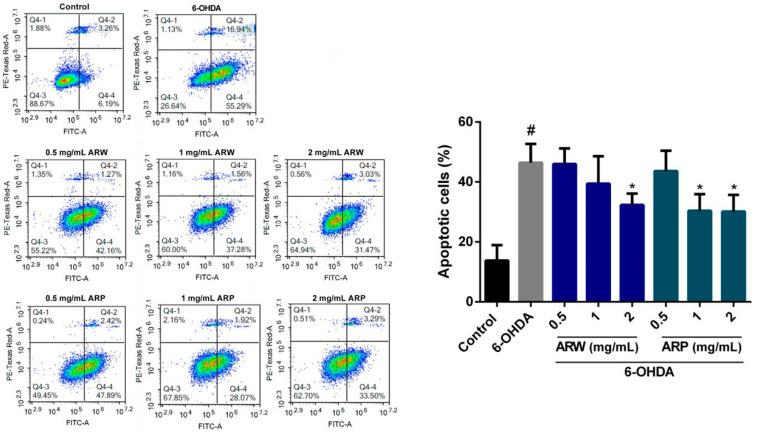
Effects of ARW and ARP treatment on 6-OHDA-induced apoptosis in SH-SY5Y cells. SH-SY5Y cells were pretreated with different concentrations of ARW and ARP for 2 h and then treated with 125 μM of 6-OHDA for 24 h. Untreated cells served as the control. The cells were stained with annexin V-FITC and PI, and the number of apoptotic cells was quantified using flow cytometry. Data are presented as a percentage relative to the control group values (mean ± SD of three independent experiments). # *p* < 0.05 indicates a statistically significant difference compared to the control group. * *p* < 0.05 indicates a statistically significant difference compared to the 6-OHDA-treated group.

The anti-apoptotic effects of ARW and ARP on SH-SY5Y cells were investigated using annexin V-FITC/PI double labeling and flow cytometry. Treatment with 125 μM of 6-OHDA resulted in an increase in the proportion of apoptotic cells from 13.77 ± 5.16% to 46.40 ± 6.29% (Figure 4). However, the number of apoptotic cells increased by 32.32 ± 3.79% and 30.11 ± 5.57% when the 6-OHDA-treated cells were treated with 2 mg/mL of ARW and ARP, respectively.

### 2.6. Effects of ARW and ARP on the Expression of Apoptosis-Related Signaling Pathways and Pro/Antioxidant Enzyme Levels in SH-SY5Y Cells

**Figure 5:**
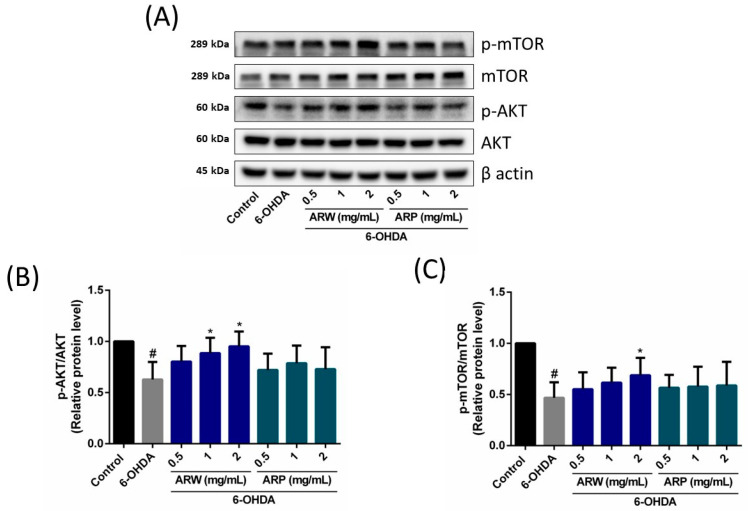
Effects of ARW and ARP treatment on the total and phosphorylated protein levels of Akt and mTOR in the SH-SY5Y cells. SH-SY5Y cells were treated with different concentrations of ARW and ARP for 2 h and then treated with 125 μM of 6-OHDA for 24 h. Untreated cells served as the control. (**A**) Protein expression levels of Akt, p-Akt, mTOR, and p-mTOR in the SH-SY5Y cells were determined using Western blotting analysis. β-Actin was used as a reference. (**B**,**C**) The amounts of the different proteins after being normalized to that of β-actin. Data are presented as the mean ± SD of three independent experiments. # *p* < 0.05 indicates a statistically significant difference compared to the control group. * *p* < 0.05 indicates a statistically significant difference compared to the 6-OHDA-treated group.

**Figure 6:**
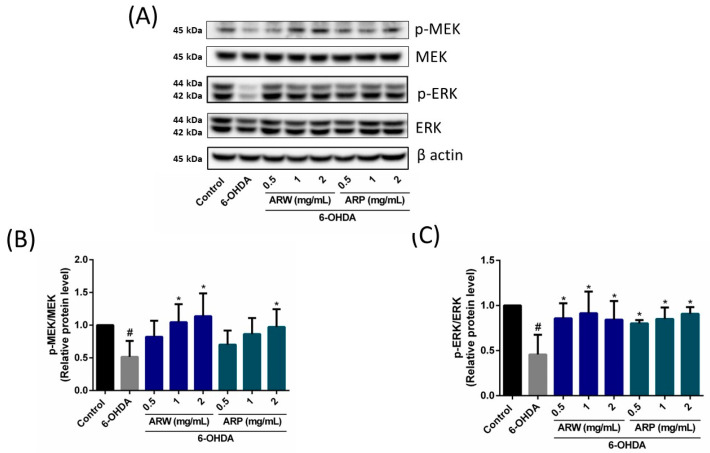
Effects of ARW and ARP treatment on the total and phosphorylated protein levels of MEK and ERK in the SH-SY5Y cells. SH-SY5Y cells were treated with different concentrations of ARW and ARP for 2 h and then treated with 125 μM of 6-OHDA for 24 h. Untreated cells served as the control. (**A**) Protein expression levels of MEK, p-MEK, ERK, and p-ERK in the SH-SY5Y cells were determined using Western blotting analysis. β-Actin was used as a reference. (**B**,**C**) The amounts of the different proteins after being normalized to that of β-actin. Data are presented as the mean ± SD of three independent experiments. # *p* < 0.05 indicates a statistically significant difference compared to the control group. * *p* < 0.05 indicates a statistically significant difference compared to the 6-OHDA-treated group.

The Akt/mTOR- and MEK/ERK-dependent signaling pathways play a significant role in the modulation of neuronal cell death. Treatment with 125 μM of 6-OHDA resulted in a substantial reduction in the protein expression ratio of phospho-Akt/Akt, phospho-mTOR/mTOR, phospho-MEK/MEK, and phospho-ERK/ERK in SH-SY5Y cells by 37.13%, 53.16%, 48.38%, and 54.28%, respectively (Figure 5 and Figure 6). The ratios exhibited a concentration-dependent increase when the SH-SY5Y cells were pre-treated with ARW (Figure 5 and Figure 6).

**Figure 7:**
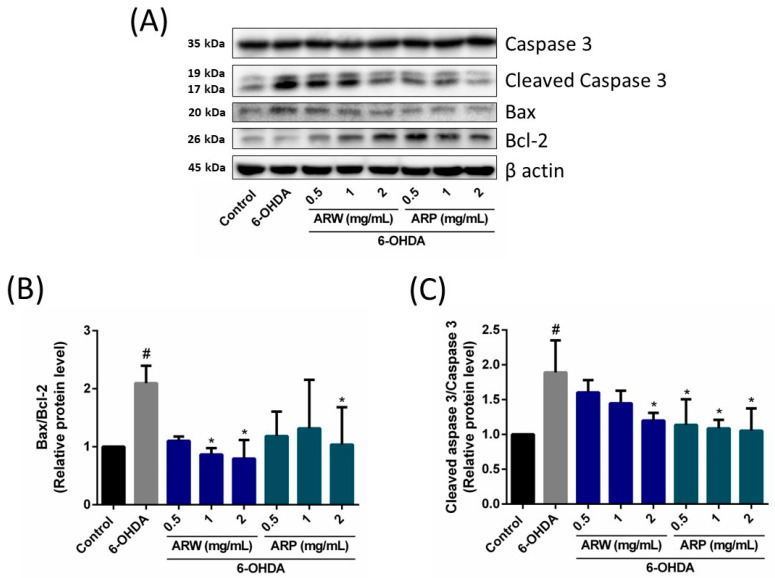
Effects of ARW and ARP treatment on the protein levels of Bax, Bcl-2, caspase 3, and cleaved caspase 3 in SH-SY5Y cells. SH-SY5Y cells were treated with different concentrations of ARW and ARP for 2 h and then treated with 125 μM of 6-OHDA for 24 h. Untreated cells served as the control. (**A**) Protein expression levels of Bax, Bcl-2, caspase 3, and cleaved caspase 3 in SH-SY5Y cells were determined using western blotting analysis. β-Actin was used as a reference. (**B**,**C**) The amounts of the different proteins after being normalized to that of β-actin. Data are presented as the mean ± SD of three independent experiments. # *p* < 0.05 indicates a statistically significant difference compared to the control group. * *p* < 0.05 indicates a statistically significant difference compared to the 6-OHDA-treated group.

**Figure 8:**
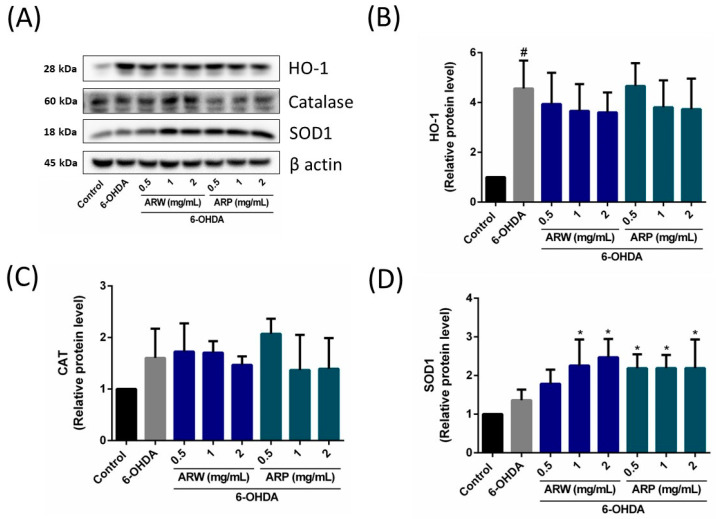
Effects of ARW and ARP treatment on the protein levels of heme-oxygenase (HO)-1, catalase (CAT), and superoxide dismutase (SOD)-1 in SH-SY5Y cells. SH-SY5Y cells were treated with different concentrations of ARW and ARP for 2 h and then treated with 125 μM of 6-OHDA for 24 h. Cells without any treatment served as the control. (**A**) Protein expression levels of HO-1, CAT, and SOD-1 in SH-SY5Y cells were determined using western blotting analysis. β-Actin was used as a reference. (**B**–**D**) The amounts of the different proteins were normalized to that of β-actin. Data are presented as the mean ± SD of three independent experiments. # *p* < 0.05 indicates a statistically significant difference compared to the control group. * *p* < 0.05 indicates a statistically significant difference compared to the 6-OHDA-treated group.

In contrast, the administration of ARP increased the protein expression ratios of phospho-MEK/MEK and phospho-ERK/ERK (Figure 6), but had no impact on the phosphor-Akt/Akt and phosphor-mTOR/mTOR ratios (Figure 5). Apoptotic protein makers including Bax, Bcl-2, and caspase 3 were also studied. 6-OHDA increased the ratios of Bax/Bcl-2 and cleaved-caspase 3/caspase 3, which could be reduced by ARW and ARP in a concentration-dependent manner (Figure 7). Furthermore, 6-OHDA treatment did not significantly alter the protein expression of SOD-1. However, treatment with ARW and ARP at 1 and 2 mg/mL enhanced the protein expression of SOD-1 (Figure 8D). Meanwhile, the protein expression of SOD-1 was increased upon treatment with 0.5 mg/mL of ARP; no such increase was observed upon treatment with 0.5 mg/mL of ARW. Neither ARW nor ARP altered the protein expression levels of heme-oxygenase-1 and catalase (Figure 8B,C).

## 3. Discussion

The neurotoxin 6-OHDA is a hydroxylated derivative of the endogenous neurotransmitter dopamine. It is commonly used to establish models of PD both in vitro and in vivo. Dopaminergic neurons internalize 6-OHDA through dopamine and norepinephrine transporters, leading to intracellular oxidation and the subsequent release of ROS such as hydrogen peroxide, superoxide, and hydroxyl radicals [9,10,11]. A substantial increase in ROS production results in a decline in the mitochondrial membrane potential and the initiation of apoptosis in PC12 cells [5]. The potential mechanism underlying the effects of the AR extract may involve the activation of the Akt/mTOR and MEK/ERK signal transduction pathways. Nevertheless, it should be noted that PC12 cells originate from rat adrenal pheochromocytomas, which may limit their ability to accurately replicate the intricacies of human neurons. Another drawback is the absence of synaptic terminals following a 14-day differentiation period, accompanied by significant morphological heterogeneity contingent on the passage number [12]. Due to the inherent constraints associated with PC12 cells, SH-SY5Y human neuroblastoma cells have emerged as an alternative in vitro neuronal model to investigate PD [13]. These cells are frequently used alongside PC12 cells to validate and compare research outcomes [14]. Consistent with prior research on PC12 cells [5], the present study demonstrated the protective effects of ARW on SH-SY5Y cells against 6-OHDA-mediated neurotoxicity. This mechanism can also be attributed to the mitigation of oxidative stress and apoptosis via the AKT/mTOR and MEK/ERK-dependent pathways. The neuroprotective efficacy of the AR extract is compelling as it has been observed to yield consistent results across two distinct neuronal cell cultures.

Another primary objective of this study was to identify the principal bioactive constituents responsible for the neuroprotective effects of AR. The chemical composition of AR was determined using analytical methods, indicating the presence of sterols, flavonoids, fatty acids, esters, aromatic acids, esters, phenols, polysaccharides, and triterpenes [15]. An earlier investigation conducted using liquid chromatography-mass spectrometry (LC-MS) showed that ARW contains several major compounds, namely, ganoderic acid A, ganoderic acid D, ganoderic acid J, oleamide, (9Z, 12Z)-octadeca-9,12-dienamide, uridine, guanosine, cytosine, uracil, adenosine, adenine, gallic acid, dextrose, and l-arabinopyranose [8]. The administration of oleamide effectively mitigated the detrimental effects of 3-NP on mitochondrial function and cell survival in rat cortical sections. The potential underlying mechanism may involve the concurrent activation of CB1R and CB2R, leading to an increase in antioxidant activity. This combined effect may arise from the preservation of mitochondrial functional integrity [16]. The molecular dynamics simulation results also indicated that ganoderic acid A has the potential to effectively target Leucine-rich repeat kinase 2 (LRRK2) [17]. LRRK2 is strongly associated with neurological illnesses, particularly PD, as certain genetic variations in this gene have been found to increase susceptibility to PD. In addition, the application of ganoderic acid A resulted in a notable enhancement of SOD activity and stabilization of the mitochondrial membrane potential in hippocampal neurons [18]. However, none of the compounds tested, except gallic acid, showed antioxidant effects in our study.

A literature-based study posited that gallic acid is a potential therapeutic agent for various neurological diseases and disorders including Alzheimer’s disease, PD, stroke, sedation, depression, psychosis, neuropathic pain, anxiety, memory loss, and neuroinflammation [19]. Gallic acid mitigates the impairment of the mitochondrial membrane potential and decreases intracellular ROS levels and apoptosis triggered by 6-OHDA in SH-SY5Y cells, as observed in in vitro experiments [20]. In SH-SY5Y cells, gallic acid decreased the ratio of the pro-apoptotic Bax protein to the anti-apoptotic Bcl-2 protein [21]. Treatment with 6-OHDA also upregulated the expression of caspase 3 and Keap 1 and downregulated the expression of Nrf2, BDNF, and p-CREB. However, these effects were effectively reversed by pre-treatment with gallic acid [21]. In vivo studies have shown that the oral administration of gallic acid at doses ranging from 50 to 200 mg/kg over 10 days significantly improves passive avoidance memory [22]. Additionally, this treatment led to an increase in the levels of total thiols and glutathione peroxidase while simultaneously reducing the levels of malondialdehyde in both the hippocampus and striatum in a rat model of PD induced by the injection of 6-OHDA into the medial forebrain bundle [22]. Furthermore, gallic acid administration enhanced motor dysfunction and increased gamma wave power in animal models of PD. These animals were subjected to a surgical procedure involving the implantation of a bipolar wire electrode in the left globus pallidus nucleus [23]. In our current investigation, the concentration of ARW that demonstrated a neuroprotective effect was 0.5 mg/mL, corresponding to a gallic acid content of 3.5 µM. Consistent with the results of previous studies, the present study showed that this concentration of gallic acid can scavenge DPPH [20]. Our findings demonstrate that this concentration of gallic acid did not significantly reduce SH-SY5Y cell death induced by 6-OHDA. However, our findings are incongruent with those of a previous investigation, wherein it was demonstrated that the induction of SH-SY5Y cell death by 50 µM of 6-OHDA may be mitigated through the pre-treatment of cells with 0.5 µg/mL of gallic acid (equivalent to 2.9 µM) for 24 h [21]. However, the potential contribution of gallic acid to the neuroprotective effect of AR should not be completely neglected, as the observed discrepancy could be attributed to the elevated concentration (100 µM) of 6-OHDA used in our current investigation.

The antioxidant and neuroprotective properties of polysaccharides derived from the Ganodermataceae family have been demonstrated in various studies. For example, polysaccharides derived from *G. lucidum* could decrease the expression of pro-inflammatory cytokines generated by lipopolysaccharides or amyloid-beta in BV-2 and primary murine microglia. In addition, these polysaccharides increased the expression of anti-inflammatory cytokines in both cell types. Furthermore, polysaccharides derived from *G. lucidum* reduced the migration of microglial cells associated with inflammation as well as morphological changes and likelihood of phagocytosis [24]. In addition, the administration of *G. lucidum* polysaccharides enhanced the viability of rat cortical neuronal cells cultivated in vitro, protecting them against oxidative stress generated by hypoxia/reoxygenation [25]. Furthermore, *G. lucidum* polysaccharides exhibited a potential neuroprotective effect on primary cultured rat cortical neurons under oxygen- and glucose-deprived conditions. This effect was achieved by downregulating caspase 3 activation and reducing the Bcl-2/Bax ratio, ultimately reducing neuronal apoptosis [26]. In addition, the oral administration of *G. lucidum* polysaccharides at various doses (100, 200, and 400 mg/kg) resulted in a significant reduction in the area of cerebral infarction [26]. This treatment also mitigated functional neurological impairment and decreased neuronal death in the ischemic cortex of rats subjected to middle cerebral artery occlusion. A fraction of oligosaccharides obtained from the mycelia of *G. lucidum* inhibited kainic acid-induced convulsions in rats [27]. This fraction mitigated the degeneration pattern observed in the CA3 region of rats, decreased astrocytic reactivity, and reduced the expression of IL-1β and TNF-α induced by kainic acid. In addition to *G. lucidum*, the neuroprotective benefits of *Poria cocos* polysaccharides have been observed in rats with Alzheimer’s disease induced by d-galactose and aluminum trichloride. These effects are attributed to the ability of *P. cocos* polysaccharides to mitigate oxidative stress, apoptosis, and inflammation and suppress the MAPK/NF-κB pathway [28].

To confirm the role of polysaccharides in the neuroprotective properties of AR, we generated a polysaccharide-enriched extract, ARP, via ethanol precipitation. This technique enables the precipitation of macromolecules such as DNA, mRNA, proteins, and polysaccharides while facilitating the removal of smaller molecules such as triterpenes and phenolic compounds. ARP is characterized by higher concentrations of polysaccharides and lower levels of triterpenes, phenolic compounds, and proteins than those in ARW. The antioxidant activity of ARP has a higher DPPH scavenging effect than that of ARW. The neuroprotective efficacy of ARP at 0.5 mg/mL surpassed that of ARW at equivalent concentrations. Similarly, our study demonstrated the protective efficacy of 2 mg/mL of ARP against the toxic effects of juglone on *C. elegans*, which was higher than that of ARW. These findings suggest that the polysaccharides present in AR have neuroprotective properties. One intriguing discovery was that ARP could upregulate MEK/ERK-dependent pathways, whereas ARW affected both the Akt/mTOR-dependent and MEK/ERK-dependent pathways. One potential explanation for this phenomenon is the role of gallic acid in the activation of the Akt/mTOR-dependent pathway [29], which is eliminated during the ethanol precipitation process during the preparation of ARP. Another noteworthy discovery that has not been previously documented is the ability of ARP to enhance the expression level of SOD-1; this effect was greater than that of ARW. This phenomenon has also been observed in polysaccharides derived from various species of the Ganodermataceae family [26,30,31].

In contrast to gallic acid, polysaccharides are macromolecules with limited ability to traverse the blood–brain barrier. This presents a potential obstacle to the utilization of polysaccharides for therapeutic purposes in brain diseases [32,33]. Hence, further investigation is necessary to determine the optimal molecular size range. One potential strategy is to employ chromatography or varying ethanol concentrations to separate polysaccharide fractions with distinct molecular sizes. In cases where the molecular sizes of the active polysaccharides exceed the optimal thresholds for passing through the blood–brain barrier, a potential approach to explore is using a nasal spray delivery system. This route of administration, known as nose-to-brain delivery, offers the advantage of circumventing the blood–brain barrier and facilitating the direct transport of therapeutic agents into the brain [34].

As a natural product, AR has several advantages. It exhibits rapid growth and can be easily cultivated, resulting in low production costs. The typical AR dose in traditional Chinese medicine is 10–15 g/decoction [15]. No adverse effects or toxicities of AR have been reported based on long-term medical and dietary records. In addition, an in vivo investigation revealed that Sprague–Dawley rats administered a single dose of AR mycelial powder as high as 2 g/kg did not experience any alterations in body weight or pathological modifications in several organs [35]. Moreover, the viability of PC-12 cells was not reduced when exposed to 2 mg/mL of aqueous AR extract [5]. The findings of this study also indicated that the administration of 2 mg/mL of ARW and ARP did not significantly affect the lifespan of *C. elegans*. Therefore, AR is considered safe.

The involvement of oxidative stress in neurodegenerative diseases is well-established. Numerous empirical studies have provided evidence supporting the potential efficacy of dietary antioxidants in the prevention and treatment of neurodegenerative disorders [36]. An example of a well-known edible and medicinal fungus in Asia is *G. lucidum*, which is claimed to enhance overall well-being and longevity as per traditional Chinese medicine. The administration of a *G. lucidum* extract could potentially improve parkinsonism induced by MPTP and protect dopaminergic neurons from oxidative stress in mice [37]. A rodent model also demonstrated that *G. lucidum* protects against mitochondrial malfunction and mortality in hippocampal neurons [38]. This effect was attributed to the antioxidant and free radical-scavenging activities of *G. lucidum*. AR is a distinct fungal species belonging to the Ganodermataceae family; however, its pharmacological properties have seldom been investigated. As previously documented [5], the present study corroborates the finding that AR exhibits promising neuroprotective effects due to its inherent antioxidant characteristics.

## 4. Materials and Methods

### 4.1. Chemicals and Reagents

Dulbecco’s modified Eagle’s medium (DMEM), fetal bovine serum (FBS), 4′,6-diamidino-2-phenylindole (DAPI), penicillin-streptomycin, and 0.25% (*w*/*v*) trypsin containing 1 mM ethylenediaminetetraacetic acid, CM-H_2_DCFDA, 5,5′,6,6′-tetrachloro-1,1′,3,3′-tetraethylbenzimidazolocarbocyanine iodide (JC-1), annexin V-conjugated fluorescein isothiocyanate (FITC), and propidium iodide (PI) were procured from Invitrogen (Carlsbad, CA, USA). The reagents 6-OHDA, 2,2-diphenyl-1-picryl-hydrazyl-hydrate (DPPH), dimethyl sulfoxide, 3-(4,5-dimethylthiazol-2-yl)-2,5-diphenyltetrazolium bromide (MTT), ganoderic acid A, ganoderic acid D, ganoderic acid J, oleamide, (9Z, 12Z)-octadeca-9,12-dienamide, uridine, guanosine, cytosine, uracil, adenosine, adenine, gallic acid, dextrose, and l-arabinopyranose were obtained from Sigma-Aldrich (St. Louis, MO, USA). A bicinchoninic acid assay kit was acquired from Boster Biological Technology Co. Ltd. (Pleasanton, CA, USA). All antibodies used for Western blotting were procured from Cell Signaling Technology (Danvers, MA, USA). Before use, all compounds were dissolved in suitable solvents and stored at −20 °C to preserve their chemical stability.

### 4.2. Preparation of ARW via Reflux Distillation and of ARP via Ethanol Precipitation

AR was provided by Hong Kong Ganoderma Center Limited (Hong Kong SAR, China). The fruiting bodies of AR were desiccated and subsequently ground into a fine powder. To prepare ARW, the powdered sample was dissolved in distilled water at a 1:20 (*w*/*v*) ratio and heated at 95 ± 2 °C for 60 min. The supernatant was collected, and the sample residue was re-extracted twice following the steps described above. Subsequently, all the extracts were pooled, filtered, and concentrated using a rotary evaporator.

ARP was prepared from ARW via ethanol precipitation, with a final ethanol percentage of 80%. Finally, the ARW and ARP were lyophilized using a freeze-dryer, and the samples were stored at −20 °C until use. Before conducting the experiments, the thawed extracts were filtered through 0.22 µm membrane filters.

### 4.3. Measurement of Chemical Content

The extracts were dissolved in 5 mL of distilled water, then 30 mL of 95% ethanol was slowly added with stirring. The resulting samples were shaken and refrigerated at 4 °C overnight. Following centrifugation, the resulting supernatant was collected to quantify the amounts of phenolic compounds, triterpenes, and proteins using a previously described methodology [5,39]. The standards used for the chemical analysis of polysaccharides, phenolic compounds, flavonoids, and triterpenes were glucose (GE), gallic acid (GAE), quercetin (QE), and oleanolic acid (OA), respectively.

### 4.4. DPPH Assay

The SC of the samples was measured using a DPPH assay. Briefly, 5 μL of each sample was mixed with 195 μL of a 24 mg/L DPPH solution and placed in each well of a 96-well plate. The reaction was performed in the dark for 60 min. Vitamin C served as the positive control. The absorbance of the reaction mixture was then measured at 515 nm using a microplate absorbance reader. The SC_50_ value was defined as the concentration of the extract that effectively scavenged 50% of the free radicals.

### 4.5. Strains, Maintenance, and Synchronization of Caenorhabditis elegans

The *C. elegans* strain used in this study was the wild-type N2 variety. Briefly, the worms were cultivated in a nematode growth medium supplemented with *Escherichia coli*. The nematode was fed with the bacterial strain OP50 (obtained from the Caenorhabditis Genetics Center at the University of Minnesota, Minneapolis, MN, USA) and maintained at 20 °C in an incubator.

Age synchronization in worms was achieved by isolating eggs from gravid hermaphrodites. The eggs were lysed in a lysis solution containing 5 M NaOH and 5% NaOCl. The mixture was vortexed for 10 min and centrifuged at 1800 rpm for 2 min. Subsequently, the liquid portion was discarded and the solid residue was washed in sterile water before being centrifuged for another 2 min. After removing the water, the remaining eggs were reconstituted in an M9 buffer to facilitate hatching. The larvae were maintained post-hatching in S medium supplemented with *E. coli*.

### 4.6. Survival Assay for C. elegans under Juglone-Induced Oxidative Stress

Wild-type N2 worms at the age-synchronized L1 larval stage were divided into six groups. The worms were then treated with 1 or 2 mg/mL of ARW, ARP, or water (control) diluted in S medium and bacteria. Following 48 h of treatment at 20 °C, the pro-oxidant juglone, a naphthoquinone derived from *J. regia*, was added at 80 μM. The worms were then incubated for 24 h at 20 °C. Subsequently, the number of surviving and deceased worms were counted.

### 4.7. Longevity Assay for C. elegans

A population of N2 worms at the age-synchronized L4 larval stage was inoculated onto NGM agar plates supplemented with an *E. coli* OP50 lawn. The worms were treated with ARW or ARP at 1 or 2 mg/mL or with water (as the control). The worms were cultivated at 20 °C and observed using a stereomicroscope (Motic SMZ-171, Nanjing, China). Daily counts of the worms were recorded from the initial day of their transfer to the experimental NGM plates (day 0) until the death of all individuals. Worms were classified as deceased when they did not respond to a delicate stimulus using a platinum wire and displayed no discernible movement in their pharyngeal pumping activity. Worms that exhibited internally hatched progeny or extruded gonads were censored and eliminated from the experimental analysis. The experiment was conducted with a minimum of 100 worms per group.

### 4.8. Cell Culture

Human neuroblastoma SH-SY5Y cells were obtained from the American Type Culture Collection (Manassas, VA, USA). These cells were cultured in DMEM supplemented with 10% FBS, 100 U/mL of penicillin, and 100 µg/mL of streptomycin. The cells were maintained at 37 °C in a humidified atmosphere containing 5% CO_2_. The medium was replaced every other day. The cells underwent differentiation through a 48-h incubation with 10 µM retinoic acid before conducting subsequent studies.

### 4.9. Cell Viability Assay

Cell viability was assessed using MTT assays following the procedure provided by the manufacturer. In brief, the spent culture medium was removed, and the cells were incubated with a working solution of MTT (0.5 mg/mL) for 4 h at 37 °C. Subsequently, dimethyl sulfoxide was added to facilitate cell lysis and the dissolution of the violet formazan crystals formed inside the cells. The absorbance was measured at 570 nm using a microplate reader.

### 4.10. Detection of Intracellular ROS Levels

Intracellular ROS were detected using CM-H_2_DCFDA staining. After drug treatment, SH-SY5Y cells were washed twice with cold PBS and stained with 2 μM of CM-H2DCFDA at 37 °C for 15 min. After washing with PBS, the stained cells were examined via flow cytometry (BD Biosciences), detecting 10,000 cells in each sample. The data were analyzed using FlowJo software (version 10.4).

### 4.11. Annexin V-Fluorescein Isothiocyanate (FITC)/PI Staining

After drug treatment, the SH-SY5Y cells were washed twice with cold PBS. The SH-SY5Y cells were then suspended in a binding solution and stained with annexin V-FITC and PI (1.0 mg/mL) for 20 min. The stained cells were promptly evaluated via flow cytometry, recording 10,000 events for each sample. The data were analyzed using FlowJo software.

### 4.12. Western Blot Analysis

Crude protein was extracted from the SH-SY5Y cells using a lysis buffer containing 1% phenylmethylsulfonyl fluoride and 1% protease inhibitor. The lysates were centrifuged at 12,500× *g* for 20 min at 4 °C, and the supernatants were collected. The bicinchoninic acid assay was used to measure the total protein concentration. Equivalent quantities of protein samples were then subjected to sodium dodecyl sulfate-polyacrylamide gel electrophoresis (SDS-PAGE) and subsequently electrotransferred onto polyvinylidene difluoride (PVDF) membranes. The PVDF membranes were blocked with a 5% nonfat milk solution in Tris-buffered saline (TBS) containing 0.1% Tween-20 for 1 h. The membrane was then incubated overnight at 4 °C with primary antibodies targeting mTOR, phospho-mTOR (Ser2448), Akt, phospho-Akt (Ser473), ERK1/2, phospho-ERK1/2 (Thr202/Tyr204), MEK, phospho-MEK (Ser217/221), superoxide dismutase (SOD)-1, catalase, heme-oxygenase-1, or β-actin. After washing with PBS, the membranes were incubated with the corresponding horseradish peroxidase-conjugated secondary antibodies for 1 h at 25 °C. After three washes with PBS, the proteins were visualized using enhanced chemiluminescence. The protein bands were recorded, and quantitative analysis of the band intensity was conducted using Syngene G: BOX Chemi XR5 (Frederick, MD, USA).

### 4.13. Data and Statistical Analysis

Data are presented as the mean ± standard deviation (SD) derived from a minimum of three independent experiments. Statistical analyses were conducted using one-way analysis of variance (ANOVA), followed by Tukey’s multiple comparison test for cases involving two or more groups. All statistical analyses were performed using GraphPad Prism software (version 6.0; GraphPad Software Inc., San Diego, CA, USA). Statistical significance was set at *p* < 0.05.

## 5. Conclusions

The current study demonstrated that the aqueous extract of AR has antioxidant and neuroprotective properties in a cellular model of PD, specifically 6-OHDA-induced apoptosis in the SH-SY5Y cell line. The mechanism of action involves the upregulation of the Akt/mTOR- and MEK/ERK-dependent pathways. The neuroprotective action of AR can be attributed to its principal active components: gallic acid and polysaccharides. The results of our study provide compelling evidence for the possible utilization of AR and its constituent components in the prevention and management of neurodegenerative disorders such as PD.

## Figures and Tables

**Table 1:** Effects of ARW and ARP in the *Caenorhabditis elegans* lifespan assay.

Treatment	Mean Lifespan (Day) ± SD	Maximum Lifespan (Day)	*p*-Value (vs. Control) *	Number of Worms
Control	15.24 ± 5.11	28		120
1 mg/mL ARW	14.61 ± 5.36	33	0.4508	137
2 mg/mL ARW	14.25 ± 4.99	28	0.2022	100
1 mg/mL ARP	14.89 ± 5.72	30	0.9632	125
2 mg/mL ARP	14.64 ± 5.82	33	0.5977	121

* The survival rate was statistically compared between the treatment groups and control by log-rank (Mantel–Cox) tests followed by the Gehan–Breslow–Wilcoxon test.

## Data Availability

Data supporting the findings of this study are available from the corresponding author, George Pak-Heng Leung, upon request.
